# Targeted panel sequencing for refining B-cell lymphoma diagnosis: a real-life, reference center experience

**DOI:** 10.1007/s00428-026-04428-y

**Published:** 2026-02-04

**Authors:** Julia Böck, Katja Maurus, Julia Doll, Stephanie Brändlein, Qunpei Yang, Katrin S. Kurz, German Ott, Ioannis Anagnostopoulos, Andreas Rosenwald, Alberto Zamò, Elena Gerhard-Hartmann

**Affiliations:** 1 Institute of Pathology, University of Würzburg, Würzburg, Germany; 2 Comprehensive Cancer Center Mainfranken, Würzburg, Germany; 3 Robert-Bosch-Hospital, Dr. Margarete Fischer-Bosch Institute of Clinical Pharmacology, Stuttgart, Germany

**Keywords:** B-cell non-Hodgkin lymphoma, NGS, Mutation, Routine diagnostics

## Abstract

The reliable diagnosis of one of the many types of B-cell lymphoma (BCL) currently requires an integrated approach comprising morphological expertise, immunophenotyping, and inclusion of clinical data, but may also incorporate flow cytometry, cytogenetics, and clonality analysis. In recent years, several studies have elucidated the mutational landscape of BCL, which may also serve as a complementary diagnostic tool. We have developed a custom next-generation sequencing panel for application in the routine diagnosis of BCL based on available literature and our diagnostic questions. We applied this panel to 160 cases of BCL or with this differential diagnosis (DD) in our routine workflow to gain further diagnostic support or on clinical request. Evaluable results were obtained in all but two cases of the entire cohort. Diagnostically informative molecular genetic profiles were identified in 72% of the evaluable cases. Focusing on 21 challenging cases with the DD of Burkitt lymphoma (BL) and the germinal center B-cell-like subtype of diffuse large B-cell lymphoma (DLBCL), we detected at least one mutation in all cases, and in 18/21 (86%) cases, panel sequencing provided significant decision guidance. In conclusion, although morphology and immunohistochemistry remain the backbone of diagnosis, panel sequencing provided substantial diagnostic assistance in many cases. It has been particularly useful in providing additional arguments to clarify the clinically important DD between BL and DLBCL in challenging cases.

**Supplementary Information:**

The online version contains supplementary material available at 10.1007/s00428-026-04428-y.

## Introduction

B-cell lymphomas (BCL) account for the majority of lymphoid neoplasms in the Western world. Currently, the World Health Organization classification of haematolymphoid tumors (WHO-HL) and the International Consensus Classification (ICC) define > 30 mature BCL subtypes [1, 2]. In view of their clinical presentation, BCL are often further categorized as aggressive or indolent. Although the diagnosis of the most common aggressive and indolent BCL in the Western world, namely diffuse large B- cell lymphoma (DLBCL) and follicular lymphoma (FL), is often straightforward, there are many constellations in which an accurate diagnosis of lymphoma can be challenging even for specialized haematopathologists. In recent years, the advent of next-generation sequencing (NGS) has led to the rapid acquisition of knowledge regarding the genomic landscape of numerous neoplasms, including BCL, which may also serve as a complementary diagnostic tool. Some lymphoma entities, such as hairy cell leukemia (HCL) and lymphoplasmocytic lymphoma (LPL)/Waldenström macroglobulinaemia (WM), show a single characteristic mutation, but mostly, there is a spectrum of common but not ultimately entity-defining mutations [3, 4]. Despite the fact that a considerable number of studies have contributed to the delineation of the mutational landscape of BCL in recent years, the number of studies that describe the application of NGS panel sequencing in a real-world, routine setting to BCL formalin-fixed paraffin-embedded (FFPE) tissue samples remains limited [5–8]. We have developed a custom NGS panel for application in the routine diagnosis of BCL based on the available literature and our diagnostic questions. The panel was designed to i) provide high informative value relative to cost, ii) include mutations with diagnostic or prognostic/predictive value, iii) ensure rapid turnaround time, and iv) enable the analysis of individual samples. Here, we report on the application of this targeted NGS panel sequencing approach for diagnostic support and, in some cases, on clinical demand in a routine diagnostic setting in 160 samples of BCL or cases with this differential diagnosis (DD) from two German reference centers for haematopathology.

## Patients and methods

### Case selection and evaluation

From October 2021 to March 2024, we performed targeted NGS analysis on a subset of 160 BCL patient samples sent for expert diagnosis to the reference centers in Würzburg and Stuttgart, Germany, to obtain diagnostic support or, in some cases, on clinical request. All cases were reviewed by expert haematopathologists (AR, AZ, EG-H, GO, IA, and KSK). Histopathological diagnoses were performed according to the WHO-HL [1, 9]. The majority of cases were tested immediately after the completion of standard diagnostic procedures (morphology, immunohistochemistry (IHC), and, in a subset of cases, fluorescence in-situ hybridization (FISH) and/or clonality studies). Case selection was not entirely random, since NGS analysis was primarily applied to challenging cases in which morphological, IHC and FISH data did not allow definitive resolution of DD. In some cases, a clinical question was specifically addressed. Ethics approval was granted by the Ethics Committee of the University of Würzburg. Further clinical information, e.g., follow-up or treatment was unavailable in most cases.

### IHC and FISH analysis

Histologic sections (2 µm) were cut and stained with hematoxylin & eosin (HE), Giemsa, and periodic acid Schiff (PAS) for routine histologic evaluation. Immunohistochemical stainings for diagnostic purposes were performed using FFPE tissue slides according to the manufacturers’ instructions and standard protocols on an automated immunostainer (BOND-III, Leica Biosystems, Nussloch, Germany).

FISH for *MYC*, *BCL2* and *BCL6* was performed using break-apart probes according to the manufacturers’ instructions and standard protocols (Zytovision, Bremerhaven, Germany). 11q aberration was analyzed using a SPEC 11q gain/loss triple color probe according to the manufacturers’ instructions (Zytovision).

### DNA extraction and clonality analyses

DNA was extracted from FFPE tissue using the Maxwell RSC Blood DNA Kit (Promega, Walldorf, Germany). DNA was quantified by quantitative PCR using the TaqMan RNase P Detection Reagents Kit (Life Technologies, Darmstadt, Germany). Inclusion criteria for further processing via NGS were DNA concentration of ≥ 0.5 ng/µl (with total DNA quantity of 10 ng) and tumor cell content/cell population of interest ≥ 10%.

B-cell receptor rearrangement analyses were performed according to the Euroclonality/BIOMED-2 protocol [10].

### NGS panel sequencing

The primary BCL panel contains 41 genes selected based on specific criteria, such as entity-specific mutation frequencies, minimization of co-mutated genes, inclusion of recurrent hotspots, functional domains, and clinically valuable predictors of prognosis, therapy response or resistance. To minimize the size of the main panel and to optimize its integration into our routine diagnostic NGS pipeline, we adopted a modular approach and designed an additional panel to encompass the entire exonic regions of the genes *ATM*, *KMT2C*, and *KMT2D*. Mutations in these genes can be widespread and therefore require sequencing of the entire coding regions, significantly increasing the size of the library. Therefore, the additional panel was only performed in certain cases, particularly when the primary panel was not considered to be sufficiently informative. Panel designs were conducted with the Ion AmpliSeq Designer software (v7.48). Supplementary Table S1 displays all covered genes of both panels with the respective genomic coordinates. Library preparation was performed with the Ion AmpliSeq Library Kit Plus according to the manufacturers recommendations. The Ion OneTouch 2 and Ion OneTouch ES automated systems or the Ion Chef System were used for templating and enrichment, followed by sequencing on the Ion S5 Plus System. Data were analyzed with the Torrent Suite Software (v5.18) and the Ion Reporter Software (v5.18/v5.20) (Thermo Fisher Scientific, Waltham, MA, USA).

### Data evaluation

Analyzed data were filtered for exonic non-synonymous variants, splice site variants in the flanking intronic regions, and small insertions/deletions with an allelic fraction ≥ 2%. Variants with a minor allele frequency of ≥ 0.2% in the general population listed in gnomAD database, ExAC database, and dbSNP database were excluded from further analysis. Moreover, to remove technical artifacts (e.g., fixation or PCR artifacts), all variants with a coverage < 200 × and an allele fraction < 5% were visualized by the Integrative Genomics Viewer (v2.15.4) [11]. Variants were classified as likely oncogenic (class 4) and oncogenic (class 5) or variants of unknown significance (VUS; class 3) using various databases (including ClinVar, CKB, cBioPortal, OncoKB, IARC database on TP53 gene variants related to cancer [12], and COSMIC) and the current literature. Molecularly uncharacterized frameshift and nonsense mutations in tumor suppressor genes were classified as loss of function mutations and therefore as likely oncogenic variants. Supplementary Table S2 and Supplementary Table S3 summarize all class 5/4/3 variants per case detected with the primary BCL panel and, if carried out, the extended panel.

## Results

### Patient and sample characteristics

We successfully analyzed 158/160 tissue samples of mature B-cell neoplasms or lymphoproliferations with this DD, comprising 30 cases of aggressive BCL, 110 cases of small cell/indolent BCL, and 18 cases with the DD of a reactive condition with our targeted sequencing approach. The series comprised a broad spectrum of tissue samples, including lymph node (LN) excisional biopsies, but also small biopsies (including core needle biopsies), surgical specimens and biopsies from the gastrointestinal tract as well as bone marrow samples, and others (Table 1 and Supplementary Table S4). A detailed overview of the mutational profile of each patient sample is given in Supplementary Table S4.

**Table 1:** Patient/sample characteristics

Total number	160
Successfully sequenced	158
Sex	
Male	91
Female	63
not specified	4
Age (years)	
Range	5–89
Median	64
Biopsy site & type	
Lymph node (excisional/large biopsy)	69
Lymph node (core/small biopsy)	5
Bone marrow	27
Gastrointestinal tract	13
Other	39
Not available	5
Diagnostic group	
Aggressive B-cell lymphoma	30
Indolent/small B-cell lymphoma	110
DD reactive condition	18

DD: Differential diagnosis

### Performance and data quality

**Fig. 1:**
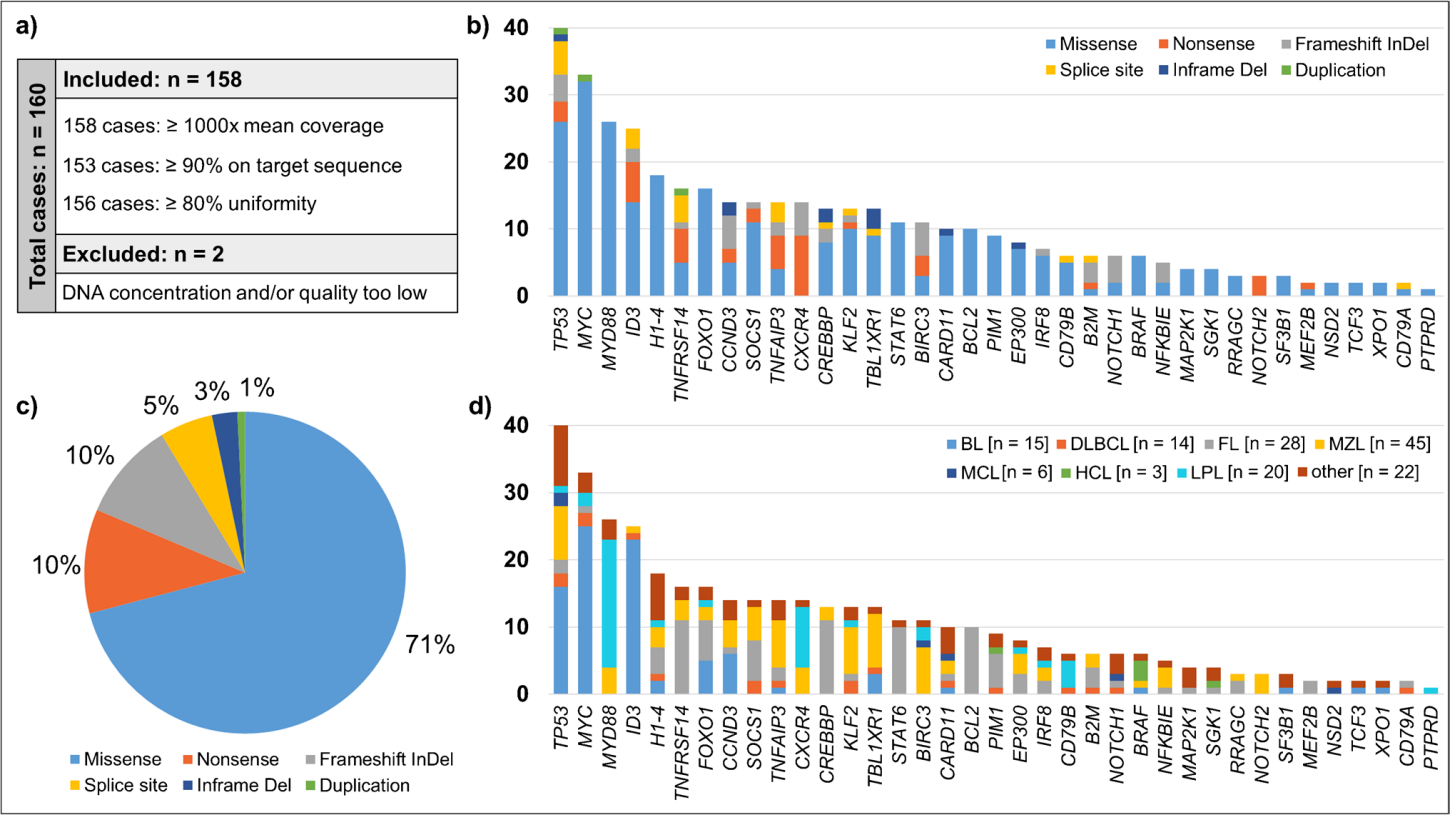
Panel-based next generation sequencing of B-cell lymphomas (primary panel): Panel performance and mutation characteristics. **a)** Quality outcome; **b)** Number of variants per mutation type for each analyzed gene; **c)** Proportion of mutation types; **d)** Number of variants per B-cell lymphoma subtype for each analyzed gene. All class 5/4/3 variants detected in this study were counted. Genes without detectable class 5/4/3 variants are not illustrated (Fig. 1b & d). Cases with a final diagnosis of a reactive condition are not included in Fig. 1d

After DNA quantification, 159/160 samples were deemed appropriate for further processing. The quantity of all prepared libraries was sufficient for sequencing. Across all sequenced samples, mean coverage was 3611 reads, mean reads on target was 97%, and mean uniformity was 91%. Defining a threshold for quality control after sequencing, 100% of cases exhibited a mean coverage of ≥ 1000-fold, 96% (153/159) of cases showed ≥ 90% reads on the target sequence, and 98% (156/159) of cases ≥ 80% uniformity. None of the processed samples failed more than one quality criterion and had to be excluded based on sequencing quality. However, four cases had many fixation artifacts, one of which had to be excluded for this reason. The other three cases were classified as validly assessable due to the higher tumor cell content (> 30%) and by using a higher threshold of 10% allelic fraction (Fig. 1a and Supplementary Table S5).

### Mutational data and contribution to diagnosis

In total, we detected 391 somatic variants with our custom B-cell panel sequencing approach, which could be classified as likely oncogenic and oncogenic variants (class 4 and 5) or VUS (class 3). Class 4 and 5 variants were most commonly found in the *TP53*, *ID3*, *MYD88* and *CXCR4* gene, whereas class 3 variants were most frequently detected in *MYC*, *H1-4*, *TBL1XR1*, and *SOCS1* in this series (Supplementary Table S6). We detected no class 3/4/5 variants in *BTK*, *EZH2, IDH1* and *PLCG2*. In total, we detected one or more mutation in 77% of the cases. Counting all variants per gene as one alteration, we found only one altered gene in 30 samples (19%), while 41 (26%) showed two, 21 (13%) three, 15 (9%) four, 11 (7%) five, 2 (1%) six, 1 (0.6%) eight, and 1 (0.6%) eleven altered genes. Two or more concomitant variants per sample were most frequently detected in the *ID3, MYC, SOCS1, KLF2* and *TP53* gene. In 36 out of 158 samples (23%), no class 3, 4 or 5 variants could be detected. However, in 10/36 cases without detectable mutations, a DD of a reactive condition was considered.

Most class 3/4/5 variants were missense variants (71%), followed by nonsense and frameshift (each 10%), as well as splice site (5%) variants (Fig. 1b and 1c). In individual genes, only missense variants were detected, most of which are entity-specific hotspot mutations such as p.L265P in *MYD88*, which is typical for LPL, p.V600E in *BRAF* as the characteristic mutation in HCL, or p.D419/p.E377 in *STAT6,* which is typical for FL (Fig. 1b and 1 d). Tumor suppressor genes such as *TP53*, *ID3*, *TNFAIP3,* or *TNFRSF14* also showed truncating mutations and/or in-frame deletions or small duplications.

Figure 1 illustrates panel performance and quality parameters, types of mutations per gene, and variant frequency per gene in the different entities. Overall, diagnostically informative molecular genetic profiles were identified in 115/158 cases (72%).

### Diagnostic support of panel sequencing in the diagnosis of aggressive BCL

**Fig. 2:**
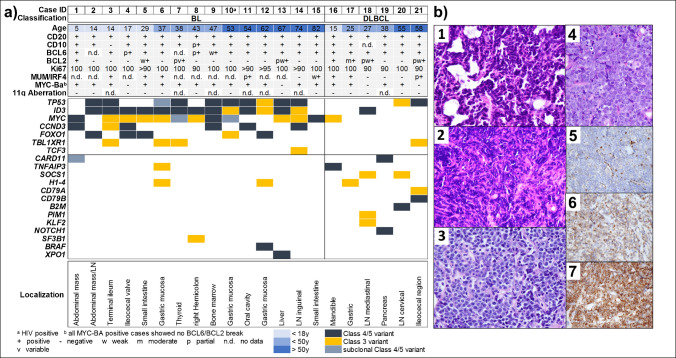
Overview of the cases with a differential diagnosis of Burkitt lymphoma (BL) and the germinal center B-cell-like (GCB) subtype of diffuse large B-cell lymphoma (DLBCL). **a)** Clinical data, immunophenotype, cytogenetics and mutational profile per case. **b)** Diagnosis was complicated due to tissue alteration (**1/2**: Case 13/18; HE, 60 × objective), borderline cytomorphology (**3/4**: Case 7/6; HE, 60 × objective), unusual immunophenotype (**5/6/7**: variable BCL2 expression within different areas of case 7, 60 × objective), or undetectable *MYC* rearrangement (not shown). Genes that are considered more indicative of BL are grouped in the upper third of the gene list (above the grey horizontal line), and genes more suggestive of DLBCL are listed below. *MYC*-BA: *MYC* FISH using a break apart probe; LN: lymph node.

We performed panel sequencing in 30 cases of aggressive BCL. The largest group of 21 cases represents cases in which the distinction between BL and germinal center B-cell-like subtype of DLBCL (DLBCL-GCB) was challenging due to impaired histological evaluation (e.g., crush and/or fixation artifacts), unusual morphological or immunophenotypical features, or undetectable *MYC* rearrangement by FISH using a break apart probe. The two cases without detectable *MYC* rearrangement that were ultimately classified as BL showed otherwise typical BL features, such as 100% proliferation (Ki-67) and a typical immunophenotype, with the only exception being weak and partial BCL2 expression in case 13, which was deemed still compatible with BL. However, we cannot rule out cryptic *MYC* rearrangements in these cases [13, 14]. In all cases, we detected at least one mutation. In 18/21 cases (86%), panel analysis provided significant diagnostic support. The most common variants in the cases finally classified as BL were *ID3* (13/15), *MYC* (12/15), *TP53* (10/15) and *CCND3* (6/15). All cases with a detectable *MYC* rearrangement showed no additional *BCL6* and/or *BCL2* rearrangement, and no 11q aberration was detected in the evaluable samples. Figure 2 provides an overview of the cases investigated with a DD of BL and DLBCL-GCB regarding mutational profile, cytogenetics, and clinical as well as histopathological and immunohistochemical features.

Panel analysis was performed in 10 additional cases of aggressive BCL with unusual features but more diverse diagnostic questions (e.g., in six cases with a DD of DLBCL with (partial) Cyclin D1 expression vs. pleomorphic mantle cell lymphoma (MCL)). Overall, the panel provided diagnostic support in 6/10 cases (60%) and was supportive in 5/6 cases involving the DD between pleomorphic MCL and DLBCL with (partial) Cyclin D1 expression, with a mutational spectrum including *ATM* mutations in both cases finally classified as MCL, but in none of the cases classified as DLBCL

### Role of panel sequencing in the differential diagnosis of indolent/small BCL and reactive conditions

We performed panel sequencing in 110 indolent/small cell BCL cases and 18 cases with a DD of indolent/small BCL and a reactive condition. The most frequently posed question within the cohort of indolent/small cell B-cell lymphoma (BCL) concerned the diagnosis of atypical FL vs. marginal zone lymphoma (MZL) (n = 33). These represent cases with unusual morphological, immunohistochemical and cytogenetic features, e.g. BCL2-/t(14;18)-negative FL, FL with diffuse growth or with marginal zone differentiation. Another frequent question, which we sought to clarify by panel sequencing, pertained to the DD of LPL/WM from MZL (n = 29), especially in cases where a lymph node involvement by LPL appeared possible. In addition, there were several cases with an unusual phenotype (e.g., (partial) co-expression of CD5 and/or CD23) with a DD of LPL in which we conducted panel sequencing. In 9 cases, panel sequencing was performed for *MYD88* mutational analysis upon clinical request. Moreover, mutational analysis of *TP53* was clinically requested in three CLL/SLL (chronic lymphocytic leukemia/small lymphocytic lymphoma) and four MCL cases, respectively.

**Fig. 3:**
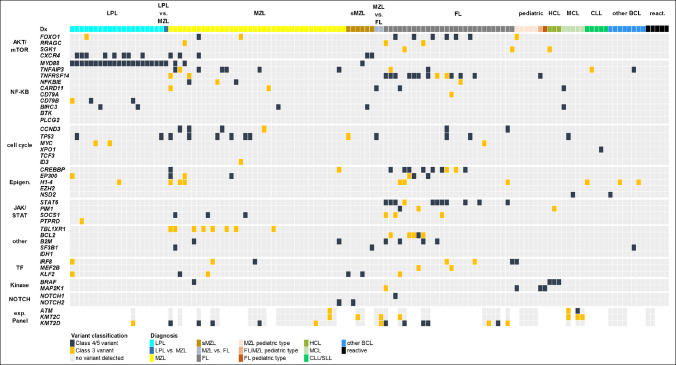
Panel sequencing results of both custom panels in the differential diagnosis of small B-cell lymphoma (BCL). LPL: lymphoplasmacytic lymphoma; MZL: Marginal zone lymphoma; sMZL: splenic Marginal zone lymphoma; FL: Follicular lymphoma; HCL: Hairy cell leukemia; MCL: Mantle cell lymphoma; CLL: Chronic lymphocytic leukemia

Figure 3 displays the sequencing results of both custom panels for the samples finally classified as FL, MZL, LPL, MCL, CLL/SLL, and HCL, as well as reactive conditions.

To evaluate the potential diagnostic value of the detected mutational profile, we searched the literature for each individual case. However, the supplemental table by deLeval et al. provides an excellent overview [3]. Supplementary Table S7 is adapted from Supplemental Table 1 by deLeval and coworkers illustrating the most commonly detected mutations across major groups of small BCL.

In all cases of LPL and HCL, we detected known hotspot mutations in the *MYD88* and *BRAF* gene, respectively. In 8/20 (40%) cases finally classified as LPL, we detected co-mutation of *CXCR4* and *MYD88*. Three cases were ultimately classified as most consistent with (S)MZL despite detection of a *MYD88* mutation. However, only one case exhibited the classical *MYD88*-L265P mutation. Furthermore, all three cases harbored additional mutations not typical for LPL, and none demonstrated plasmacytic/secretory differentiation. No mutations and no clonal B-cell population were detected in any case finally classified as a reactive condition. However, in one case we favored a reactive condition despite an *IGHV* rearrangement but recommend haematological examination. In the DD of FL vs. MZL, our custom B-cell panel provided diagnostic support in 25/33 (76%) cases. In total, we detected a significantly higher number of mutations in the cases finally classified as FL compared to MZL cases (p = 0.009; t-test). The most common class 3/4/5 variants in FL affected *TNFRSF14* (12/28), *STAT6* (11/28), *CREBBP* (9/28) and *FOXO1* (6/28). Moreover, we applied our B-cell panel to 28 small B-cell neoplasms with various diagnostic questions. These included cases with prominent plasmacytoid/plasmacellular differentiation and a DD of a plasma cell neoplasia, unusual BCL with co-expression of CD5 as well as cases with a DD of a pediatric-type FL/MZL and occasionally cutaneous BCL (Supplementary Table S4).

Furthermore, the extended panel was conducted in an additional 55 cases with the DD of MZL, FL, MCL, LPL, and reactive conditions. In 21 cases, a class 3/4/5 variant was detected in *ATM* (4/55), *KMT2C* (7/55), and *KMT2D* (14/55) (Fig. 3). However, extended panel sequencing revealed an oncogenic variant in only one out of 26 cases without genetic alterations in the primary B-cell panel. In total, a diagnostically informative mutational profile was identified in 45% of the cases in which extended panel sequencing was performed.

## Discussion

This study presents real-world data on our customized NGS BCL panel sequencing approach, which was applied to 160 BCL cases to obtain further diagnostic support or upon clinical request. Although a wealth of NGS data in BCL has been published, few studies have investigated its use in a routine diagnostic setting [5–8, 15]. With an overall success rate of 99%, we underline the technical feasibility of NGS panel analysis in routine FFPE samples with high sample diversity, consistent with our previously published results using a targeted panel sequencing approach in T- and NK-cell lymphomas [16]. The 41 plus 3 genes in our BCL-focused modular custom panel approach were selected based on the available literature and our diagnostic or clinical questions. Overall, diagnostically informative molecular genetic profiles were obtained in 72% of all cases with this approach, which is in the range of the data published by Bommier et al. and Pillonel and coworkers in a routine setting [5, 8]. However, the value of NGS panel sequencing as a supportive measure varies depending on the specific diagnostic question being considered. Furthermore, as with the assessment of morphology and some immunomarkers, a degree of subjectivity cannot be excluded as to whether a particular mutation/mutational profile is diagnostically supportive, and the absence of mutation(s) may also confer diagnostic value.

In the group of aggressive BCL, the major reason for conducting panel sequencing in this series was to provide diagnostic support in the DD between BL and DLBCL-GCB through the mutational profile. The DD between BL and DLBCL-GCB can be challenging based on morphological, immunophenotypical, and cytogenetic data alone and often has a significant clinical impact. BL is genetically characterized by a *MYC* translocation to the immunoglobulin loci, usually identified by FISH [17]. However, the *MYC* translocation can rarely be „cryptic “ and undetectable by standard FISH break-apart probe approaches [13, 14]. Moreover, about 10–15% of DLBCL also show a *MYC* translocation [18, 19]. Furthermore, the immunophenotype between DLBCL-GCB and BL can be similar, leading to cytomorphology as the ultimate discriminator. However, the latter may include a degree of subjectivity and can be difficult or even impossible to assess in small and/or altered specimens (e.g., due to poor fixation or crush artifacts). Moreover, some BL cases may exhibit larger cell nuclei and a higher degree of pleomorphism, making the distinction from DLBCL based on cytomorphology difficult. These diagnostic challenges have also been reflected in the now abandoned concept of BCL-U (B-cell lymphoma unclassifiable, with features intermediate between BL and DLBCL), whereby most of these cases seem closer to BL according to their mutational profile [20]. In our series, panel sequencing provided additional diagnostic arguments in most cases in which the DD of BL and DLBCL was challenging due to inferior morphological assessment because of crush or fixation tissue artifacts, „borderline “ cytomorphology, unusual immunohistochemical features or undetectable *MYC* rearrangement. In these difficult cases the final diagnosis is typically based on a Bayesian approach that integrates successive data; in this context, the detection of mutations which are known to be preferentially present in BL shifted the probability in favor of BL. The most commonly mutated genes in the cases finally classified as BL in our series were *ID3*, *MYC, TP53,* and *CCND3* mutations. *ID3* is a negative regulator of the transcription factor *TCF3*, and inactivating *ID3* mutations are known to be common in BL, but rare in DLBCL [21–23]. Likewise, the additionally observed *CCND3* mutations in 6/15 cases classified as BL also bear diagnostic value favoring BL [23]. However, it must be underlined that the key to a meaningful classification are not single mutations, but rather the mutational profile in conjunction with the clinical, morphological, immunophenotypical and cytogenetic data in each individual case.

An uncommon but diagnostically relevant question is the distinction between pleomorphic MCL and DLBCL with cyclin D1 expression and/or *CCND1* rearrangement. Recent studies [24, 25] indicate that *CCND1* rearrangements in DLBCL are usually acquired late, often involving switch regions, and are associated with a mutational profile distinct from MCL, showing almost no mutations in *ATM* and displaying a profile more similar to ABC-DLBCL.

Looking at samples with a DD of specific indolent/small BCL and reactive conditions, panel sequencing also provided diagnostic support in the majority of cases. In cases in which the DD encompassed entities with characteristic mutations, such as the *MYD88* L265P mutation in LPL/MW or the *BRAF* V600E mutation in HCL, the value of panel sequencing was – not surprisingly- particularly high.

B-cell panel sequencing was also employed to assist in the DD of FL with unusual features and (nodal) MZL. Although the diagnosis of FL and MZL is straightforward in most cases, it can be occasionally challenging based on morphological, immunohistochemical and cytogenetic features, particularly regarding BCL2-/t(14;18)-negative FL, FL with diffuse growth or marginal zone differentiation. Thus, our cohort is biased towards an enrichment of this type of FL, which is probably reflected in the mutational profiles. In our series, we detected a high frequency of *STAT6* mutations and common co-occurrence of *CREBBP* or *TNFRSF14* mutations in FL. In contrast, we observed a low frequency of *MEF2B* mutations and no *EZH2* mutations, which are known to be common in ‘classical’ FL [26, 27]. Overall, we found a relatively broad spectrum of mutations in this cohort, and although panel sequencing provided diagnostic support in many cases, it was often not as straightforward as in the DD between BL and DLBCL-GCB.

To further categorize BCL and/or differentiate from a reactive condition, the extended panel for the analysis of the genes *ATM*, *KMT2D*, and *KMT2C* was performed in 55 cases. In 21/55 cases, a class 3/4/5 mutation was detected. In only one of the 26 cases that exhibited no mutation in the primary B-cell panel, an oncogenic mutation was identified in the extended panel. In the present study, the analysis of these three genes contributed significantly to further classification in only a relatively small number of cases, for instance in cases where a DD included any type of unusual MCL (cyclin D1 negative, pleomorphic) was considered, since *ATM* mutations are very common in MCL and rare in other lymphoma types (with the exception of CLL). Consequently, we think that the analysis of these three genes can be effectively addressed by a separate panel, thus keeping our custom panel sequencing approach as versatile, efficient and cost-effective as possible for optimal applicability in routine diagnostics.

Regarding the detected mutation classes, we observed a relatively high number of VUS (class 3), for which functional in vitro validation and confirmation in larger patient series are warranted. However, since the current databases are not well annotated for lymphoma-associated mutations, we believe that in the present context many of these class 3 mutations may provide diagnostic information and, therefore, we included them in the presented data.

In addition to genes that may be diagnostically useful, the panel also includes prognostically and therapeutically relevant genes, such as *MYD88* and *CXCR4* for LPL or *TP53* for CLL/MCL. These genes have been lately increasingly requested by the clinical colleagues. Moreover, our custom panel includes genes with potential predictive relevance, such as *BTK* and *PLCG2*, whose mutation can convey ibrutinib resistance in CLL [28] and other B-cell lymphomas, such as MCL and LPL [29].

In conclusion, we have successfully applied a custom NGS-panel to 158 BCL cases and samples with this DD in a routine diagnostic setting. Technical performance of this panel was excellent even analyzing small/crushed biopsies, decalcified material and samples from different laboratories. Although morphology and immunohistochemistry remain the backbone of diagnosis, panel sequencing provided substantial diagnostic assistance supporting the correct diagnosis in many cases. It was particularly useful in providing additional arguments to clarify the clinically important DD between BL and DLBCL-GCB in challenging cases. However, a careful, histology-informed and context-dependent analysis of all diagnostic aspects is key for a meaningful interpretation of mutational data in BCL.

## Supplementary Information

Below is the link to the electronic supplementary material.

ESM 1 (XLSX 201 KB)

## Data Availability

Data were available from the corresponding author on request.
